# Cerebral microvascular dysfunction in metabolic syndrome is exacerbated by ischemia–reperfusion injury

**DOI:** 10.1186/s12868-017-0384-x

**Published:** 2017-09-08

**Authors:** Nathalie Obadia, Marcos Adriano Lessa, Anissa Daliry, Raquel Rangel Silvares, Fabiana Gomes, Eduardo Tibiriçá, Vanessa Estato

**Affiliations:** 1 Laboratory of Cardiovascular Investigation, Oswaldo Cruz Foundation, Avenida Brasil, 4365, Rio de Janeiro, RJ 21045-900 Brazil; 2 National Institute of Cardiology, Rio de Janeiro, Brazil; 3 Institute of Drug Technology, Oswaldo Cruz Foundation, Rio de Janeiro, Brazil

**Keywords:** Metabolic syndrome, Laser speckle contrast imaging (LSCI), Cerebral microvascular blood flow, Ischemia and reperfusion

## Abstract

**Background:**

Metabolic syndrome (MetS) is associated with an increased risk of cerebrovascular diseases, including cerebral ischemia. Microvascular dysfunction is an important feature underlying the pathophysiology of cerebrovascular diseases. In this study, we aimed to investigate the impacts of ischemia and reperfusion (IR) injury on the cerebral microvascular function of rats with high-fat diet-induced MetS.

**Results:**

We examined Wistar rats fed a high-fat diet (HFD) or normal diet (CTL) for 20 weeks underwent 30 min of bilateral carotid artery occlusion followed by 1 h of reperfusion (IR) or sham surgery. Microvascular blood flow was evaluated on the parietal cortex surface through a cranial window by laser speckle contrast imaging, functional capillary density, endothelial function and endothelial–leukocyte interactions by intravital videomicroscopy. Lipid peroxidation was assessed by TBARs analysis, the expression of oxidative enzymes and inflammatory markers in the brain tissue was analyzed by real-time PCR. The cerebral IR in MetS animals induced a functional capillary rarefaction (HFD IR 117 ± 17 vs. CTL IR 224 ± 35 capillary/mm^2^; *p* < 0.05), blunted the endothelial response to acetylcholine (HFD IR −16.93% vs. CTL IR 16.19% from baseline inner diameter *p* < 0.05) and increased the endothelial–leukocyte interactions in the venules in the brain. The impact of ischemia on the cerebral microvascular blood flow was worsened in MetS animals, with a marked reduction of cerebral blood flow, exposing brain tissue to a higher state of hypoxia.

**Conclusions:**

Our results demonstrate that during ischemia and reperfusion, animals with MetS are more susceptible to alterations in the cerebral microcirculation involving endothelial dysfunction and oxidative stress events.

## Background

Cerebrovascular diseases are characterized by brain dysfunction caused by abnormalities of the cerebral circulation, mainly alterations of the cerebral blood supply, including ischemic and hemorrhagic stroke [1] Various conditions, such as aging, hypertension, hypercholesterolemia, diabetes, obesity and atherosclerosis are known to enhance cerebrovascular risk, promote cerebral hypoperfusion and facilitate the onset and progression of cognitive impairment [2].

In this context, the ischemia–reperfusion (IR) injury plays an important role in stroke pathophysiology. It has already been shown that brain ischemia followed by reperfusion leads to a cascade of pathological mechanisms that contribute to irreversible tissue damage [3].

Metabolic syndrome (MetS) is a cluster of several risk factors for cerebrovascular diseases and type 2 diabetes. Furthermore, it is associated with insulin resistance, glucose intolerance, central body fat deposition, dyslipidemia and arterial hypertension [4]. It has been proposed that the deleterious effects of MetS on cerebral function are, at least in part, due to increased oxidative stress, neuro-inflammation, and impaired vascular reactivity with a progressive decline in microvascular density [5, 6]. These pathological adaptations damage cerebral vascular auto-regulation and blood flow reserve, leading to cognitive impairment and dementia [7, 8].

The link between inflammation and MetS is well described. Evidence suggests that inflammatory disorders can result from obesity, insulin resistance and dyslipidemia, worsening the MetS scenario [9]. Adipocyte hypertrophy leads to hypoxia in visceral adipose tissue, which is considered a causative factor of inflammation [10]. This pro-inflammatory state contributes to the secretion of several cytokines by adipocytes, such as interleukin-6 (IL-6) and tumor necrosis factor (TNF-α), interfering with intracellular insulin signaling and compromising endothelial function [11, 12]. This common pro-inflammatory and pro-thrombotic state may explain the association between MetS and thrombotic/ischemic events, such as thromboembolic stroke and transient ischemic attack, mainly due to the central role of atherosclerotic plaque development [13] in stroke events [14, 15].

It is well established that hypertension and diabetes are important modifiable risk factors for stroke by increasing the risk of cerebral hypoperfusion and ischemia and decreasing the oxygen supply to the brain [6, 16]. Moreover, it has been shown that type 2 diabetes mellitus is often associated with endothelial vascular dysfunction [7]. Under homeostatic conditions, the endothelium maintains normal vascular tone and blood fluidity, which offers protection against pro-inflammatory factors. However, during a chronic inflammatory process, such as MetS and type 2 diabetes mellitus, the endothelium undergoes a loss of vasodilator and anti-thrombotic factors and an increase in vasoconstrictor and pro-thrombotic products [17]. These vascular alterations increase endothelial damage and accelerate the rate of atherosclerotic plaque formation and consequent atherothrombosis, culminating in ischemic events. Vascular endothelial dysfunction is an important feature of MetS, and it is probably related to oxidative stress and insulin resistance [18].

The microcirculation is an important site of damage in most target organs of cardiovascular disease, such as the brain, heart and kidneys. The functional alterations in the small arteries, arterioles, and capillaries are the basis of target organ damage. In this context, the present study focuses on the impact of MetS on brain microcirculation, which leads to an increase in susceptibility to cerebral damage during cerebral IR. For this purpose, we used an experimental model of cerebral ischemia and reperfusion in rats with MetS induced by a high-fat diet.

## Results

### Hemodynamic and metabolic effects of high-fat diet-induced metabolic syndrome in rats

Table 1 summarizes the characteristics of the rats in the CTL or HFD groups without the IR procedure. We observed a marked increase in the systolic blood pressure and heart rate after 20 weeks of the diet in the HFD compared to the CTL group. There was no significant difference in body weight between the CTL and HFD groups. The HFD groups exhibited increased fasting plasma glucose, insulin levels and the HOMA-IR parameter (Table 1).

**Table 1:** Hemodynamic and metabolic effects in high-fat diet-induced metabolic syndrome in rats

	CTL-sham	HFD-sham
Body weight (g)	428 ± 29	410 ± 46
SAP (mmHg)	116 ± 3	144 ± 2***
HR (bpm)	332 ± 9	429 ± 5***
Plasma fasting glucose (mg/dL)	102 ± 7	228 ± 15***
Plasma fasting insulin (ng/mL)	2.5 ± 0.5	6.5 ± 1.1**
HOMA-IR	12.5 ± 3.6	55.8 ± 13.4*

Values represent mean ± standard error, n = 8

*SAP* Systolic arterial pressure, *HR* heart rate, *HOMA-IR* homeostasis model assessment of insulin resistance

*** *p* < 0.001, ** *p* < 0.01, * *p* < 0.05 versus CTL-sham

### Effects of ischemia and reperfusion on cerebral microvascular blood flow

**Fig. 1:**
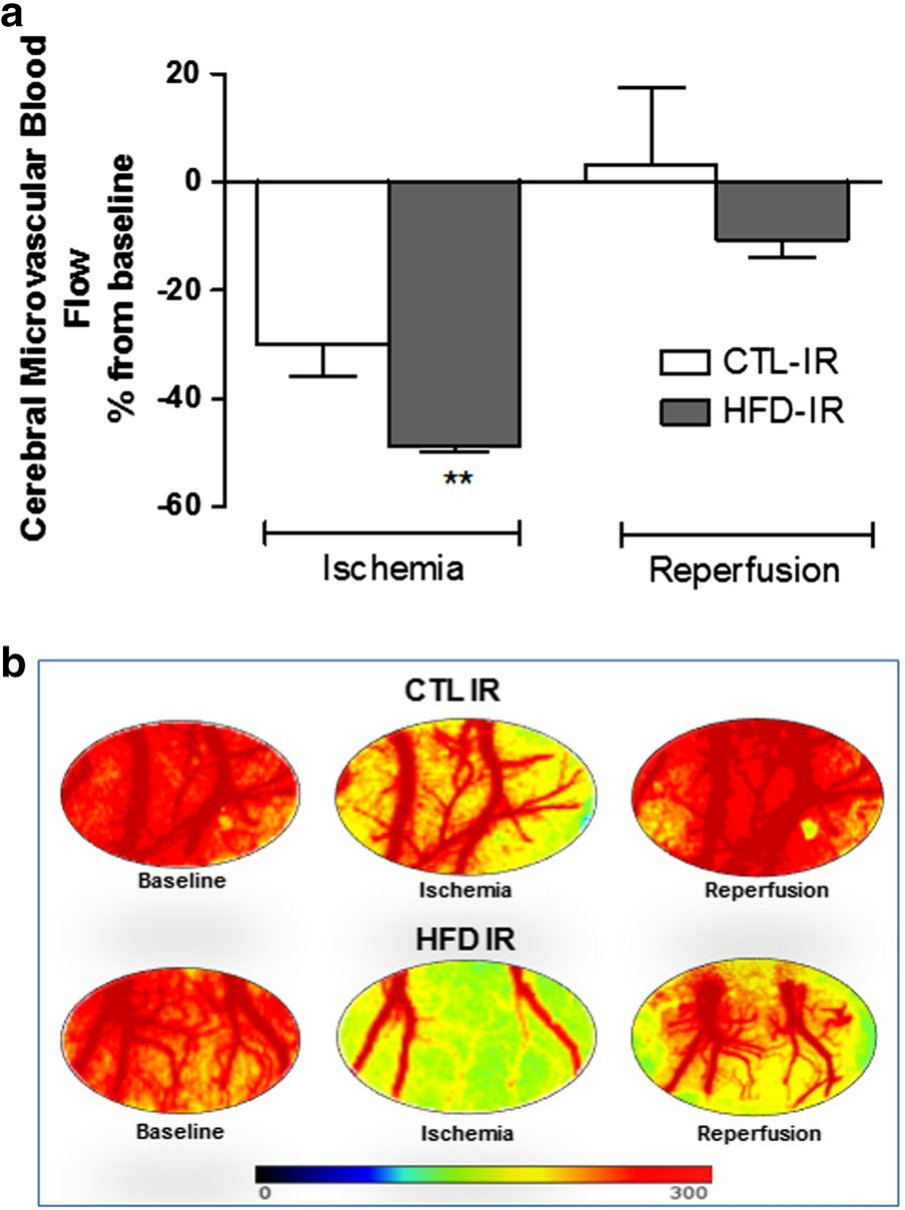
Cerebral microvascular blood flow assessed by laser speckle contrast imaging during cerebral ischemia and reperfusion. **a** Values represent the percentage of variation from basal values related to ischemia and reperfusion. **b** Representative images of laser speckle contrast imaging of the brain cortex of rats at baseline, at 30 min of ischemia and after 60 min of reperfusion in animals fed a control diet or high-fat diet and submitted to brain ischemia and reperfusion (CTL-IR and HFD-IR). Values represent the mean ± SEM, n = 5 per group. **p < 0.01 versus basal values

The laser speckle contrast imaging revealed that the basal values of microvascular brain blood flow were significantly reduced in the HFD group (183 ± 21 APU, *p* < 0.05) when compared to CTL group (227 ± 20 APU). During the first 3 min of the occlusion period, we observed gradual flow compensation in control animals, while in HFD animals this compensation occurs sharply. We also observed that the HFD animals exhibited a marked decrease in brain microvascular blood flow during the ischemia period, which was significantly different from the control group (*p* < 0.01, Fig. 1a). During the reperfusion period, the animals of the HFD group presented altered flow recovering, although not statistically significantly different, compared with the control group (Figs. 1a, b).

### Effects of ischemia and reperfusion on brain capillary density, leukocyte–endothelial interaction and endothelial function

**Fig. 2:**
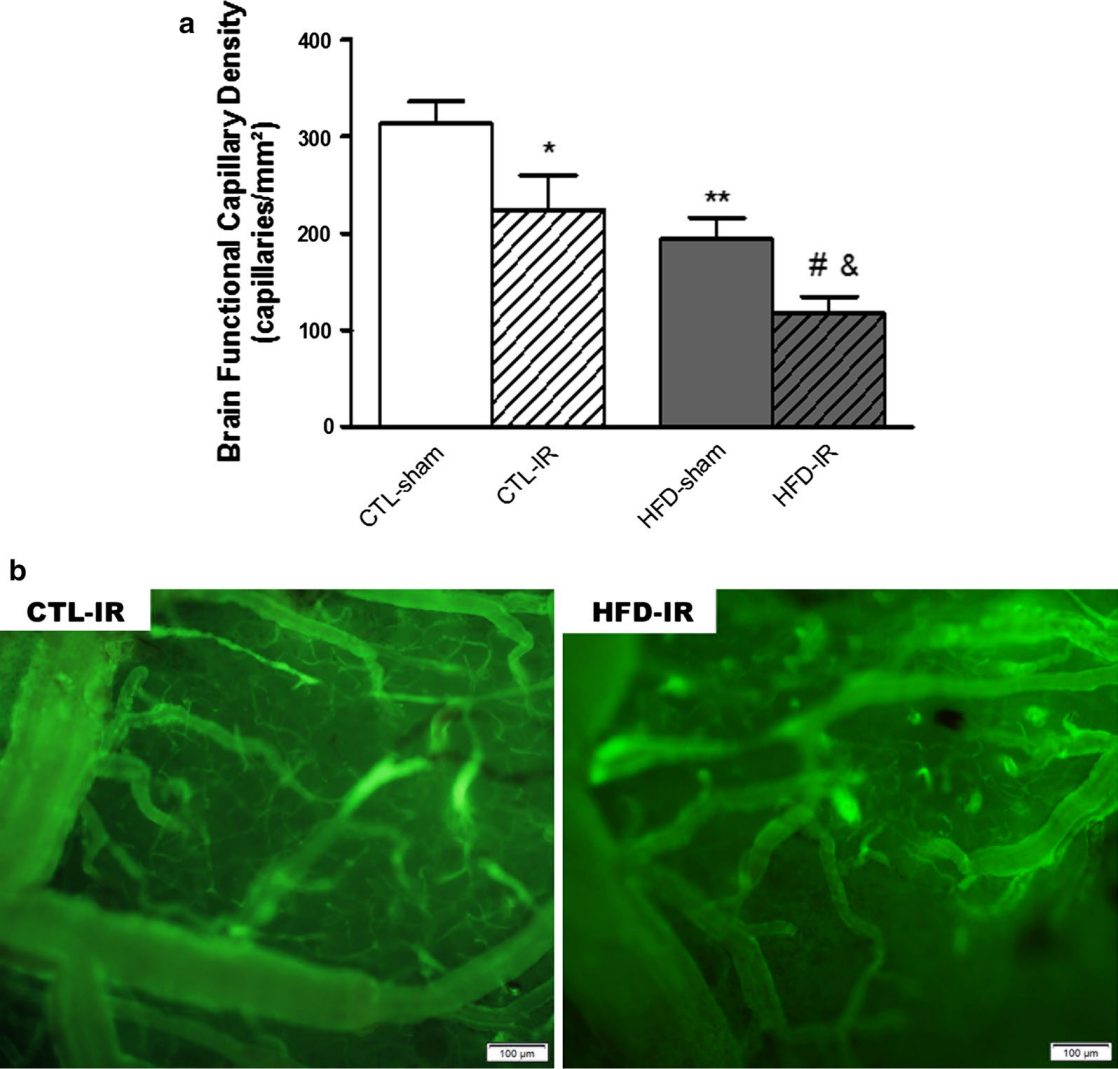
Brain microvascular functional capillary density in control or HFD animals after cerebral ischemia and reperfusion. **a** Brain functional capillary density in the cerebral microcirculation of rats with metabolic syndrome (HFD-sham) or control rats (CTL-sham) after ischemia and reperfusion (CTL-IR or HFD-IR). **b** Representative intravital fluorescence microscopic images of the effect of ischemia and reperfusion on the brain microcirculation of CTL-IR or HFD-IR rats after intravenous administration of FITC-labeled dextran (magnification ×100) Values represent the mean ± SEM. n = 8. *p < 0.05; **p < 0.01 versus CTL-sham; #*p* < 0.05 versus CTL-IR; & *p* < 0.05 versus HFD-sham

The cerebral microcirculatory parameters were evaluated after the IR or sham period and revealed that HFD-sham rats exhibited lower numbers of spontaneously perfused capillaries in their brain cortices (F(1,38) = 15.99 *p* < 0.01) than CTL-sham rats. After the IR period, the HFD-IR group presented a more pronounced functional capillary rarefaction (F(1,38) = 5.679 *p* < 0.05) when compared to both the HFD-sham and CTL-IR groups. The IR also induced a significant reduction in the number of spontaneously perfused capillaries in the CTL-IR (F(1,38) = 5.68 *p* < 0.05) group when compared to the CTL-sham (Fig. 2a, b).

**Fig. 3:**
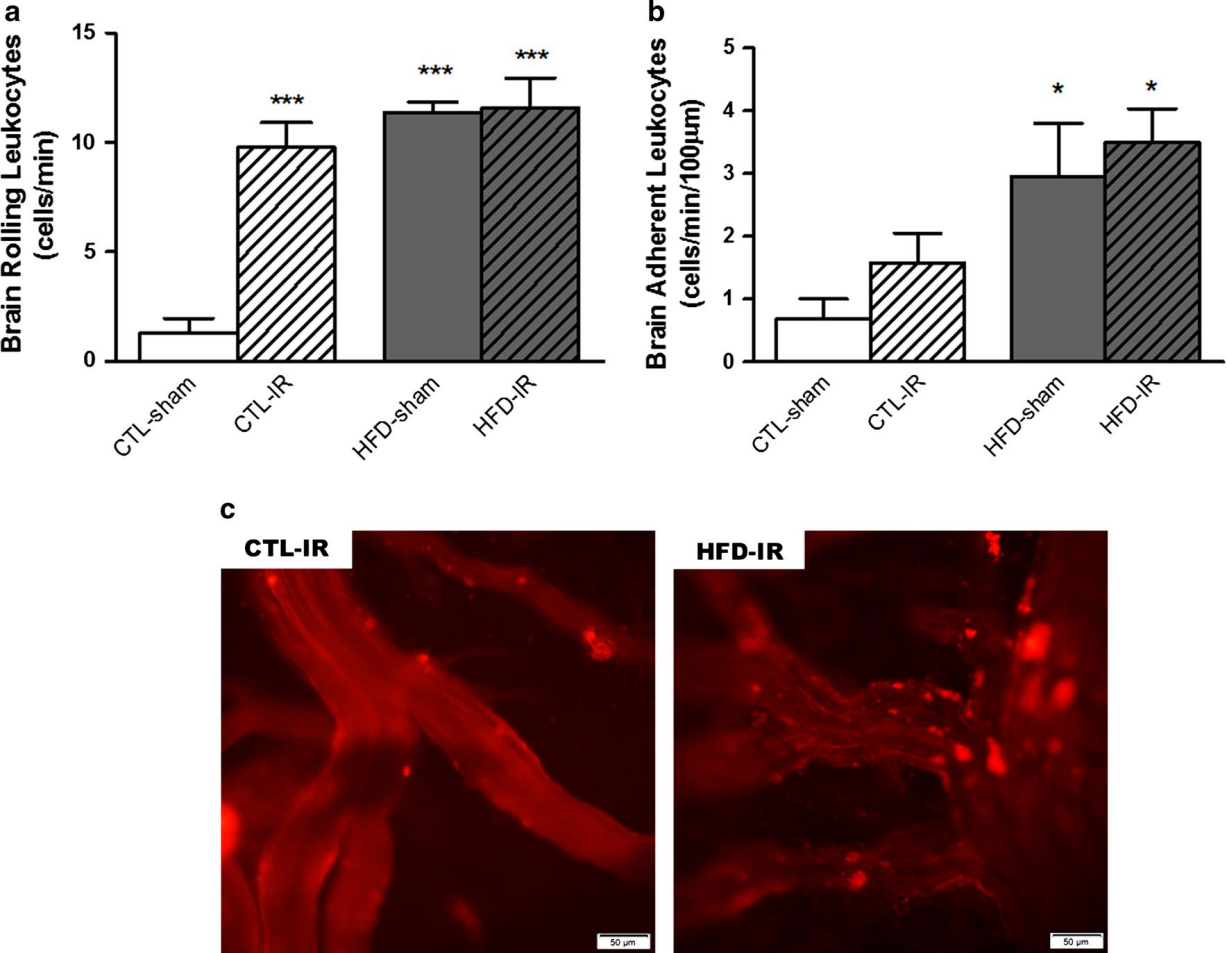
Leukocyte–endothelial interaction in brain microcirculation in control or HFD animals after cerebral ischemia and reperfusion. **a** Rolling and **b** adherent rhodamine 6G-labeled leukocytes evaluated by intravital fluorescence microscopy on the cerebral post-capillary venules of rats fed a normal or high-fat diet (CTL-sham, HFD-sham) after cerebral ischemia and reperfusion (CTL-IR or HFD-IR). **c** Representative images of the effects on the brain microcirculation in the cerebral post-capillary venules of rats after ischemia and reperfusion (CTL-IR or HFD-IR) (magnification ×200). Values represent the mean ± SEM. n = 8 per group. *p < 0.05; ***p < 0.001 versus CTL-sham

The endothelial–leukocyte interactions in the cerebral post-capillary venules evaluated by intravital fluorescence microscopy revealed that the HFD-sham group presented an increased number of rolling (F(1,25) = 43.06 *p* < 0.001) and adherent leukocytes when compared to the CTL-sham group (Fig. 3a, b). After the IR period, there was a significant increase in the number of rolling leukocytes in the HFD-IR and CTL-IR groups (F(1,25) = 23.19 *p* < 0.001) (Fig. 3a–c), while an increase in the number of adherent leukocytes was observed in the HFD-IR group (F(1,26) = 11.20 *p* < 0.01) but not in the CTL-IR group (Fig. 3b, c).

**Fig. 4:**
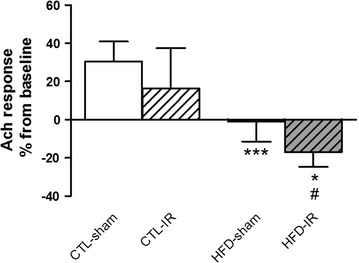
Evaluation of microvascular endothelial function by intravital fluorescence microscopy. Percentage of variation from the basal values of arteriolar inner diameter after topical acetylcholine (Ach, 10^−6^ μM) administration in the cranial window of rats fed a normal or high-fat diet (CTL-sham, HFD-sham) after cerebral ischemia and reperfusion (CTL-IR or HFD-IR). Values represent the mean ± SEM. n = 4 per group. *p < 0.05, ***p < 0.001 versus CTL-sham, #*p* < 0.05 versus CTL-IR

The evaluation of the microvascular endothelial function by topical Ach in cerebral arterioles by intravital fluorescence microscopy revealed that in the CTL-sham group, the inner diameter of cerebral arterioles increased (≈30%) from baseline, indicating preserved endothelial function. However, we observed that in the HFD-sham group, the vasodilation response to Ach was significantly impaired (≈−1.6%; F(1,11) = 20.50 *p* < 0.001) when compared to the CTL-sham group. Furthermore, after the IR period, Ach induced a vasoconstrictor response in the HFD-IR (≈−17%; F(1,11) = 4.470 *p* < 0.05) group compared to the CTL-IR group (≈16%) (Fig. 4).

### Effects of cerebral ischemia and reperfusion in oxidative stress

**Fig. 5:**
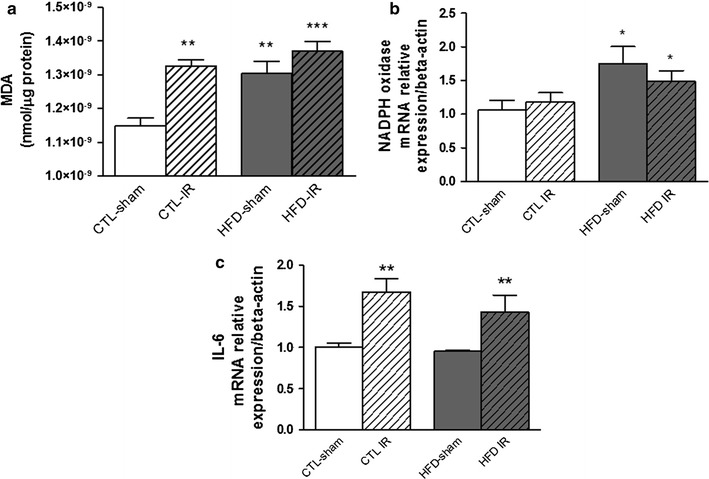
Oxidative stress, RNAm expression of NADPH and IL-6 in the brain of animals after cerebral ischemia and reperfusion. Malondialdehyde levels **a** and real-time PCR analyses of mRNA transcript levels of genes coding for the NADPH oxidase p47 subunit **b** and IL-6 **c** in the brains of rats fed a normal (CTL-sham) or high-fat diet (HFD-sham) or after cerebral ischemia and reperfusion (CTL-IR or HFD-IR). Values represent mean ± SEM, n = 6 per group *p < 0.05; ** *p* < 0.01, *** *p* < 0.001 versus CTL-sham

The brain oxidative stress was evaluated by two different methods: the analysis of the brain content of malondialdehyde (MDA) by the TBARS method, which is a standard methodology used for demonstrating the oxidative damage reflecting quite accurately the level of lipid peroxidation [19], and by the quantitative RT-PCR of the NADPH oxidase. The HFD-sham group showed a significant increase in brain MDA levels (F(1,20) = 12.69 *p* < 0.01) when compared to the CTL-sham group. The IR induced an increase in the MDA levels in the brains of CTL-IR rats that was also significantly pronounced in the HFD-IR group (F(1,20) = 18.78 *p* < 0.001) (Fig. 5a).

The Real-Time PCR was used to assess the NADPH oxidase p47 subunit and IL-6 gene expression in the brains of all studied groups. A significant increase in the NADPH oxidase expression was observed in the brains of the HFD-sham group (F(1,22) = 5.542 *p* < 0.05) when compared to the CTL-sham group, whereas this parameter was not altered in the HFD-IR group (Fig. 5b).

### Effects of cerebral ischemia and reperfusion in the IL-6 gene expression

The IL-6 gene expression was evaluated by qRT-PCR to determine the level of the pro-inflammatory cytokine in the brains of all animal groups. IL-6 gene expression was not altered in the HFD-sham group compared to the CTL-sham group. Moreover, it was equally significantly increased in the HFD-IR and CTL-IR (F(1,20) = 13.59 *p* < 0.01) groups when compared to the CTL-sham group (Fig. 5c).

## Discussion

The data reported in the present study indicate that the presence of MetS exacerbates cerebral microvascular rarefaction and endothelial dysfunction induced by ischemia and reperfusion. These effects may be related to the increase in oxidative stress and the low grade inflammatory process that results from the metabolic syndrome induced by high-fat diet intake.

Endothelial dysfunction is characterized by reduction of the bioavailability of vasodilators, particularly nitric oxide, and an increase in endothelium-derived contracting factors [20]. It is also considered to be an early marker of cardiovascular risk factors such as hypertension, diabetes mellitus, hypercholesterolemia and obesity [21], and it plays an important role in microvascular damage [22]. In our study, rats fed a high-fat diet exhibited an expected brain capillary rarefaction that could be related to the marked vascular dysfunction and insulin resistance [22].

The peripheral insulin resistance may facilitate the onset of MetS, worsening microvascular rarefaction [23]. In this study, we observed that the endothelium-dependent vasodilator response to acetylcholine in the brain cortex significantly increased the arteriole diameter in the control animals, while in the MetS animals this effect was impaired; when submitted to IR, it presented vasoconstriction, suggesting vascular dysfunction in the MetS animals. [24]. These results corroborate the studies of Chantler et al. demonstrating the pathophysiological mechanisms involved in the microvascular rarefaction of different tissues, such as skeletal and cardiac muscle, reporting that the balance between systemic vascular oxidative stress and endothelial function has a key role in the progression and severity of microvascular rarefaction [7].

The bilateral common carotid occlusion model used in this study causes cerebral damage and impaired neuronal function [25]. Some studies have demonstrated that 20 min of ischemia followed by 20 min of reperfusion induces lipid peroxidation and leukocyte adhesion within the rat’s brain. However, as longer reperfusion is required to prevent further neuronal damage, in many cases, it may further aggravate ischemic injury, such as brain cell necrosis or apoptosis, a phenomenon referred to as “cerebral reperfusion injury” [26]. This phenomenon leads to oxidative stress by increasing free radical release [27], reactive oxygen species (ROS) and playing an important role in the development of neuronal degeneration [28]. The IR was also reported to increase the microvascular permeability, leading to blood–brain barrier breakdown, an imbalance between vasodilator and vasoconstrictor factors and activation of coagulation [27, 29]. The disruption of the blood–brain barrier that occurs during the acute phase of ischemia can exacerbate brain injury, increasing the edema during the reperfusion period [30]. Diabetes also aggravates the cerebral reperfusion injuries because of the increased release of ROS and the pro-inflammatory state [31].

Our results demonstrated that animals with MetS submitted to cerebral IR showed an exacerbated decrease in brain perfusion during the ischemia period, suggesting dysfunction in the vasomotor control of the microcirculation in animals with MetS. Laser speckle technology allowed the real time observation of a marked and sudden reduction of cerebral blood flow during the ischemia period in MetS animals, exposing brain tissue to a higher state of hypoxia. In addition, although the recovery in blood flow of the MetS animals is not statistically different from the control animals, it is important to note that they still have a reduction of microvascular blood flow of around 10% when compared to their baseline values.

The adhesion of leukocytes to the vascular endothelium is a hallmark of the inflammatory process. We observed that IR induced an increase in the number of rolling leukocytes in the control and MetS animals compared to those observed in the sham-animals with MetS. This effect indicated that high-fat diet-induced metabolic syndrome and IR lead to similar levels of brain vascular inflammation. Villringer et al. were the first to demonstrate that endothelial–leukocyte interactions in the rat brain microcirculation occur after 10 min of global ischemia followed by 60 min of reperfusion [32]. Later, it was reported that the increased rolling and adherent leukocytes could be observed within the first 30 min of reperfusion after 10 min of global ischemia in rats [33]. The initial adhesive interactions between the leukocytes and venular endothelium after IR are tethering and rolling, followed by adhesion and transmigration. This process is generally regulated by the sequential activation of different families of adhesion molecules, such as P-selectin and E-selectin ICAM-1 [34], that are expressed on the surfaces of leukocytes and endothelial cells in this vascular bed. The recruited leukocytes may contribute to reperfusion injury through the release of proteases, ROS and other inflammatory mediators. The coupling of the leukocyte adhesion molecules in endothelial cells can activate signaling pathways, leading to increased amplification of the inflammatory response [35].

The post-ischemic inflammation contributes to neuronal injury and stroke development. This process is mediated by endogenous cytokines and chemokine mediators and the rolling leukocytes. Cytokines play a key role in leukocyte chemotaxis as potent chemokine inducers. Therefore, cytokines and chemokines act as primary and secondary mediators, respectively, to attract leukocytes in inflammatory conditions [35]. Activated astrocytes and microglia are also active in cytokine and chemokine release [36]. These molecules are responsible for the accumulation of inflammatory cells in the brain tissue after IR injury, such as TNF-α, IL-1β and IL-6 cytokines, initiating the inflammatory response and inducing the expression of other cytokines after IR injury [37]. Consistent with these findings, our results demonstrated that IR induced an increase in mRNA expression of IL-6 after cerebral IR in the brains of both the control and MetS animals.

It is well established that increased ROS production is one of the major causative factors for cerebral IR injury, and NADPH oxidase is a major source of ROS production [38]. NADPH oxidase present in neutrophils and macrophages is an inducer of ROS when they undergo an “oxidative burst” during inflammation, constituting the organism’s defense against pathogens and other deleterious molecules. p47 ^phox^ is one of the six enzyme subunits composing the NADPH oxidase located in the plasma membrane, and it can form an active NADPH complex when combined with several other cytosolic subunits [24]. Consistent with these findings, we observed increased NADPH oxidase expression and MDA levels in the brains of animals with MetS, either submitted or not to IR, suggesting that the oxidative stress induced by the high-fat diet-induced metabolic syndrome is comparable to that evoked by IR.

Taken together, we assume that MetS aggravates only the cerebral functional capillary rarefaction and arteriolar endothelial dysfunction parameters after the IR procedure. In the other analyzes of brain inflammation (rolling and adhesion), levels of MDA and IL-6 and NADPH oxidase gene expression, this aggravation was not observed, since animals with MetS already presented an increased vascular and oxidative inflammatory process. These results suggest that animals with MetS reach a high grade of brain vascular inflammatory activation comparable to IR injury in control animals, justifying the non-statistically significant difference in the above mentioned parameters in the HFD diet groups.

Limitations of this study should be considered. First, we performed intravital microscopy and molecular analyses at the end of the IR period; however, the microvascular blood flow analysis was performed throughout the IR period. Second, the intravital microscopy and the microvascular blood flow analysis were performed on the cerebral cortex of the animals, but all the molecular analyses were done on one brain hemisphere.

## Conclusion

Our results suggest a potential association between MetS and cerebral microvascular dysfunction involving vascular inflammation and oxidative stress events. Moreover, the experimental IR model could represent a useful tool for understanding the pathophysiology of brain IR as well as proposals for therapeutic approaches of brain injuries in MetS.

## Methods

### Animals and experimental protocol

Forty-eight male Wistar rats (300 g) supplied from the central animal facilities of the Oswaldo Cruz Foundation, Brazil, were housed in standard cages in a temperature-controlled room (22 ± 1 °C) with a 12-h light/dark cycle and water ad libitum. All experimental procedures were conducted in accordance with the internationally accepted principles for the *Care and Use of Laboratory Animals* and approved by the Oswaldo Cruz Foundation Animal Welfare Committee (license # LW-13/14).

At 8 weeks of age, the rats were randomly divided into two experimental groups and fed either a standard chow (Nuvilab-CR1, Nuvital Nutrients Ltda, Paraná, Brazil) (CTL, N = 24) or a high-fat diet (HFD, N = 24) for 20 weeks. The control diet contained 23% protein, 71% carbohydrate, 6% lipids and 1.3% NaCl. The HFD is manipulated in our laboratory and comprised of standard chow supplemented with corn starch, condensed milk, saturated animal fat (lard) and 0.5% NaCl. Regarding micronutrient composition, the CTL contained 23% protein, 71% carbohydrate and 6% lipids, and the HFD contained 14% protein, 56% carbohydrate, 30% lipids and 0.5% NaCl. This HFD was analyzed by a nutritionist, confirming the high content of lipids in the diet, and approved by the ethical committee [39]. At the end of 20 weeks, the minimum necessary period to induce metabolic changes by the abovementioned diet [39], the animals were divided into the following experimental groups: rats fed with HFD submitted or not to 30 min of cerebral ischemia through bilateral carotid occlusion followed by 1 h of reperfusion (HFD-IR or HFD-sham, respectively) or standard chow submitted or not to IR (CTL-IR or CONT-sham, respectively). All animals of the HFD group entered to the study because they presented the metabolic and hemodynamic alterations such as hypertension and hyperglycemia at the end of diet protocol. However, data and tissues obtained from the animals that died during the IR procedure were not included in the study.

### Hemodynamic measurements

Systolic arterial pressure (SAP) and heart rate (HR) was measured by a non-invasive measurement of tail pressure (Visitech Blood Pressure Analysis System, BP-2000 Apex, NC, USA). Measurements were taken at the beginning and in the 20th week of the experimental protocol.

### Surgical procedures

At the end of the diet period, the animals were randomly divided for microcirculatory analysis. The rats were anesthetized with a combination of ketamine (90 mg/kg) and xylazine (10 mg/kg, i.p.) (Cristália, SP, Brazil). The right jugular vein was catheterized to permit the injection of the anesthetic agents and fluorescent dye. The rats’ central temperature was monitored with a rectal probe, and the body temperature was maintained at 37 ± 0.5 °C using a homeothermic blanket system (Harvard Apparatus, Holliston, MA, USA). After the anesthetic and surgical procedures, both common carotid arteries were isolated, separated from the vagus nerve and clamped using a microvascular clamp (Vascu-statt No 1-531, Scalan International, Saint Paul, MN, USA) to induce cerebral ischemia for 30 min and then unclamped to provoke reperfusion for 1 h [40]. The sham animals underwent the same procedures described above, but without clamping and unclamping of the arteries.

### Cerebral intravital microscopy with epi-illumination and fluorescence

To access the cerebral microcirculation after the IR period, each rat was fixed in a stereotaxic frame, and a cranial window over the left parietal bone (1–5 mm lateral, between the coronal and lambdoid sutures) was created using a high-speed drill to expose the brain’s microvascular surface, as previously described [41]. After the intravenous administration of 0.1 mL of 2% fluorescein isothiocyanate (FITC)-labeled dextran (molecular weight 150,000; Sigma Chemical Co., St. Louis, MO, USA), the brain capillaries were observed with epi-illumination at 460–490 nm using a 520-nm emission filter, and the images were acquired with a 10× ocular and 10× objective microscope (Olympus BX51WI, NY, USA), producing a final magnification of 100× on the monitor. The capillary count was done using cellSens Standard 1.9 software (Olympus NY, USA) for 1 min/field. Only the continuously perfused capillaries were counted to determine the mean functional capillary density, expressed by the number of capillaries/mm^2^. Additionally, this window enabled visualization of in vivo leukocyte recruitment [42]. To label circulating leukocytes, 0.3 mg/kg rhodamine 6G was injected intravenously. Fluorescing leukocytes were visualized by intravital microscopy on post-capillary venules via epi-illumination at 510–550 nm using a 590-nm emission filter. The leukocyte–endothelial interaction was evaluated by counting the number of leukocytes adhering to the venular wall (100 µm long) over 30 s and was expressed as the number of cells/min/100 µm. Leukocyte rolling was defined as the movement of white blood cells into the vessel at a speed lower than the circulating red blood cells and is expressed as the number of cells/min. We determined these parameters in brain surface venules with diameters ranging from 50 to 120 μm.

### Microvascular endothelial function

The endothelium-dependent vasodilator response to acetylcholine was evaluated in cerebral arterioles using intravital microscopy; the cranial window was suffused with Ach (Ach, 10^−6^ M) for 5 min and the internal diameters of the arterioles were measured before and after substance exposure with cellSens Standard 1.9 software (Olympus, NY, USA). Vascular responses were expressed as percentage change (%) from baseline values [21].

### Laser speckle contrast imaging

Cerebral blood flow was measured by laser speckle contrast imaging (Perimed, Järfälla, Sweden), which is a technique that provides a perfusion index proportional to the concentration and mean velocity of red blood cells for monitoring microvascular blood flow in real time [43]. The cerebral microvascular blood flow was the only parameter measured during the whole IR period, allowing the observation of the micro hemodynamic alterations. The time points of image and data acquisition of cerebral microvascular blood flow, shown in Fig. 1a, b, were performed at the first minute of baseline, at the first minute of ischemia and at the end of the 60 min of reperfusion.

To access the cortical cerebral blood flow, the animal still anesthetized in the stereotactic apparatus was placed under the laser beam and a region of interest (ROI) was selected within the same cranial window used for the intravital microscopy. The animals were maintained on a stable surface in a room with a constant temperature of 25 °C and were placed under the laser light system with image contrast with a wavelength of 785 nm for the continuous measurement of tissue blood perfusion in real time. Analysis of 16 laser speckle images per second and the relative cerebral blood flow of all the animal groups during the IR period was completed using Perisoft software (Perimed, Järfälla, Sweden) and expressed as arbitrary perfusion units (APU).

### Metabolic parameters

All the metabolic analyses were performed in fasting animals. At the end of the experiments, the animals’ blood samples were collected to perform the glucose test using an automatic glucometer (One Touch Ultra 2^®^, Johnson & Johnson Medical S.A., Buenos Aires, Argentina). Insulin levels were measured by ELISA using a commercial kit (Enzyme-linked immunosorbent assay, Millipore, USA). Insulin resistance was evaluated by calculating the HOMA (Homeostasis Model Assessment index), where HOMA-IR = [insulin (U/mL) × glucose (mmol/L)]/22.5.

### Assessment of oxidative stress

Immediately after the IR period and the microcirculation analysis, all animals were euthanized by an overdose of pentobarbital and the whole right cerebral hemisphere was used for the oxidative stress and mRNA blinded analysis.

To characterize the oxidative stress in rat brains, we measured thiobarbituric acid reactive species (TBARS) [44]. The samples were collected and homogenized in cold phosphate buffer of a pH 7.4 with BHT (final concentration 0.2%). Shortly, the samples (0.5 mL) were mixed with equal volumes of thiobarbituric acid 0.67% (Sigma Chemical, St. Louis, MO) and then heated at 96 °C for 30 min. TBARS were determined by the absorbance at 535 nm. Results were expressed as malondialdehyde (MDA, ε = 1.56 × 10^5^ M^−1^ cm^−1^) per milligram of protein (BCA assay) [44].

### Endothelial NADPH oxidase and IL-6 gene expression evaluation by qRT-PCR

The total RNA was isolated using 100 µg of the right cerebral hemisphere using the RNeasy Mini Kit (Qiagen). cDNA synthesis was performed using the High Capacity cDNA reverse transcription kit (Applied Biosystems) from 1 µg of RNA in a final volume of 20 µL. The qRT-PCR was performed using the Power SYBR Green PCR Master Mix (Applied Biosystems) according to the manufacturer’s instructions in the 7500 Fast Real-Time PCR system (Applied Biosystems). The initial sequences used for PCR amplification were as follows: Nicotinamide adenine dinucleotide phosphate (NADPH) oxidase p47 subunit (phox), forward: 5′-GTGAAGCCATCGAGGTCATTC-3′ and reverse: 3′-CCCGCGGCTTCTAATCTGT-5′; IL-6 forward: 5´TCCTACCCCAACTTCCAATGC-3′ and reverse -3′TGGATGGTCTTGGTCCTTAGCC-5′; and beta-actin (β-a): forward: 5′-CCACCCGCGAGTACAACCTTCTT-3′ and reverse: 3′-GAAGCCGGCCTTGCACATGCC-5′. The relative gene expression of interest was calculated using the ΔΔCt method and normalized by beta-actin expression.

### Statistical analysis

The results were expressed as the mean ± SEM/group, and comparisons between groups considering two factors (i.e. surgery and diet) were analyzed with two-way ANOVA statistical test. We also indicated significance F values and degrees of freedom. All tests were followed by Bonferroni’s post hoc test. Comparisons between two groups were made with unpaired t tests. Differences with *p* values of less than 0.05 were considered significant. All calculations were performed using a computer-based statistical package (GraphPad Software Inc., La Jolla, USA).

## Abbreviations

Ach: acetylcholine
APU: arbitrary perfusion units
FITC: fluorescein isothiocyanate
HFD: high fat diet
HOMA-IR: homeostasis model assessment
ICAM: intercellular adhesion molecule
IL: interleukin
IR: ischemia and reperfusion
MDA: malondialdehyde
MetS: metabolic syndrome
NADPH: nicotinamide adenine dinucleotide phosphate
qRT-PCR: quantitative reverse transcription polymerase chain reaction
ROS: reactive oxygen species
SAP: systolic arterial pressure
T-BARS: thiobarbituric acid reactive species
TNF: tumor necrosis factor
